# Safety and Efficacy of Engineered Toxin Body MT-3724 in Relapsed or Refractory B-cell Non-Hodgkin's Lymphomas and Diffuse Large B-cell Lymphoma

**DOI:** 10.1158/2767-9764.CRC-22-0056

**Published:** 2022-05-05

**Authors:** Paul A. Hamlin, Vasile Musteata, Steven I. Park, Christine Burnett, Kristina Dabovic, Thomas Strack, Eric T. Williams, Banmeet S. Anand, Jack P. Higgins, Daniel O. Persky

**Affiliations:** 1 Department of Medicine, Lymphoma Service, Memorial Sloan Kettering Cancer Center, New York, New York.; 2 Institute of Oncology, ARENSIA EM, Chisinau, Republic of Moldova.; 3 Levine Cancer Institute, Charlotte, North Carolina.; 4 Molecular Templates, Inc., Jersey City, New Jersey.; 5 Molecular Templates, Austin, Texas.; 6 University of Arizona Cancer Center, Tucson, Arizona.

## Abstract

MT-3724, a novel engineered toxin body comprised of an anti-CD20 single-chain variable fragment genetically fused to Shiga-like Toxin A subunit, is capable of binding to and internalizing against CD20, inducing cell killing via permanent ribosomal inactivation. This study evaluated MT-3724 in patients with relapsed/refractory B-cell non-Hodgkin lymphoma (r/rNHL). This open-label, multiple-dose phase Ia/b trial included a dose escalation in patients with r/rNHL according to a standard 3+3 design. Primary objectives were to determine the MTD and pharmacokinetics/pharmacodynamics. In a dose expansion study at MTD in serum rituximab-negative patients with diffuse large B-cell lymphoma (DLBCL), primary objectives were safety, tolerability, and pharmacokinetics/pharmacodynamics. Twenty-seven patients enrolled. MTD was 50 μg/kg/dose with 6,000 μg/dose cap. Thirteen patients experienced at least one grade ≥3 treatment-related adverse events; the most common grade ≥3 event was myalgia (11.1%). Two patients (75 μg/kg/dose) experienced grade 2 treatment-related capillary leak syndrome. Overall objective response rate was 21.7%. In serum rituximab-negative patients with DLBCL or composite DLBCL (*n* = 12), overall response rate was 41.7% (complete response, *n* = 2; partial response, *n* = 3). In patients with detectable baseline peripheral B cells, treatment resulted in dose-dependent B-cell depletion. The proportion of patients with anti-drug antibodies (ADA) increased during treatment and the majority appeared to be neutralizing based on an *in vitro* assay; nevertheless, tumor regression and responses were observed. MT-3724 demonstrated efficacy at the MTD in this population of previously treated patients with r/rDLBCL, with mild-to-moderate immunogenic safety events.

**Significance:**

This work describes the safety and efficacy of a new pharmaceutical pathway that could provide a treatment option for a subset of patients with a critical unmet therapeutic need. The study drug, MT-3724, is capable of targeting B-cell lymphomas via a unique, potent cell-killing mechanism that appears to be promising.

## Introduction

In the United States, diffuse large B-cell lymphoma (DLBCL) accounts for approximately 28% of newly diagnosed non-Hodgkin lymphoma (NHL) cases and is the most common lymphoid malignancy in the United States (1, 2). The current standard of care for first-line therapy, R-CHOP, is curative in approximately two-thirds of patients, while the remainder are refractory to or relapse following an initial response (3). Without other curative therapeutic options, these patients have a poor prognosis, with median overall survival (OS) of 6.3 months (4). For transplant-eligible patients, the standard of care is second-line therapy, then autologous stem cell transplantation (SCT) or chimeric antigen-receptor T-cell (CAR-T) therapy, depending on chemosensitivity. These options, however, are limited by age and eligibility criteria (5, 6). Consequently, continued development of new therapeutics remains important. The surface antigen CD20 is a well-validated target in multiple disease states and CD20 expression among patients with DLBCL is typically persistent and high, representing an attractive target for therapies with new mechanisms of action (7, 8).

Engineered toxin bodies (ETB) are comprised of a proprietarily engineered form of a Shiga-like Toxin A subunit (SLT-A) genetically fused to antibody-like binding domains (9). MT-3724, a novel ETB construct comprised of an anti-CD20 single-chain variable fragment genetically fused to SLT-A, is capable of binding to and forcing internalization of CD20, a cell surface protein that does not otherwise internalize; subsequently, MT-3724 is routed to the endoplasmic reticulum and induces potent direct cell kill via permanent inactivation of ribosomes (10). Preclinical *in vitro* studies have demonstrated that MT-3724 specifically targets and directly kills CD20^+^ cells, resulting in decreased tumor growth in the Daudi-Luc xenograft murine model (11).

Importantly, unlike mAbs such as rituximab, the direct-kill mechanism by MT-3724 does not depend upon the immune system (e.g., complement activation or antibody-dependent cellular cytotoxicity). MT-3724 may thus overcome immunologic changes associated with mAb resistance or biological variations in malignant cells (12).

We herein report the results of the dose escalation and expansion portions of a phase Ia/b study of MT-3724 in patients with NHL (NCT02361346).

## Materials and Methods

### Study Design

This open-label, multiple-dose, global phase Ia/b study of MT-3724 included dose escalation with a standard 3+3 ascending dose schema and safety and tolerability assessments in patients with r/rNHL. The dose escalation design and dose range were informed by preclinical data from GLP nonhuman primate studies where the highest nonseverely toxic dose (HNSTD) was determined to be 150 μg/kg. In addition, MT-3724 only uses the subunit A of shiga-like toxin which, *in vitro*, is biologically inactive without subunit B (13).

Primary objectives of dose escalation were to determine the MTD and pharmacokinetic/pharmacodynamic profiles of MT-3724. Secondary objectives for dose escalation included safety and tolerability, pharmacodynamics, and efficacy of MT-3724.

The study also included a dose expansion phase, which was added to address a potential interaction between circulating rituximab and MT-3724 efficacy (11). This additional dose-expansion evaluated safety and efficacy of MT-3724 in serum rituximab-negative patients with DLBCL treated at the MTD. Tumor response was assessed by the International Working Group (IWG) Response Criteria for Clinical Trials (14).

The primary objectives of the dose expansion study were to assess safety, tolerability, and pharmacokinetics/pharmacodynamics profiles of MT-3724 at the MTD.

The safety analysis population consisted of all patients who signed the informed consent form and received any amount of MT-3724. The response evaluable population consisted of patients who had at least one postbaseline efficacy assessment. Patients who discontinued due to disease progression or died prior to disease assessment were included in the efficacy analysis. The pharmacokinetic analysis population was defined as all patients who received any amount of MT-3724 and for whom the on-treatment pharmacokinetic data are considered to be sufficient and interpretable.

The study protocol was designed by its sponsors in collaboration with investigators, and was approved by each site's institutional review board, independent ethics committee, or research ethics board. The study was conducted in accordance with the Declaration of Helsinki and Good Clinical Practice guidelines and was registered with ClinicalTrials.gov (NCT02361346) using CONSORT guidelines. All patients gave written informed consent. In addition, a Data Monitoring Committee (DMC) was established to review all clinical data and help determine the MTD prior to start of patient enrollment.

### Treatment

All subjects were to receive a single course of study drug via 1 hour (+15 minutes) intravenous infusion of MT-3724, on days 1, 3, 5, 8, 10, and 12 (all ±1 day) of a 21-day cycle. Following the first dose in each cycle, there was to be a minimum observation period of 21 days after dose 1 for each subject prior to initiating the next treatment cycle. Subjects were to have successfully completed a cycle if they receive at least 4 of the 6 infusions in that cycle and were followed for 21 days after the first dose in that cycle.

All doses were to be administered within the first 14 days of the 21-day cycle on days 1, 3, 5, 8, 10, and 12 (all ±1 day). The doses were to be at least 20 hours apart (more than five half-lives of MT-3724 in serum) and were not to be administered on more than two consecutive days.

Given the known risks for innate immune responses and infusion-related reactions with other immunotoxins, one premedication agent from each of the following three therapeutic classes was to be considered within 60 minutes before the start of MT-3724 infusion in each cycle:

- Oral antipyretic agent, e.g., acetaminophen
- Intravenous H1 histamine receptor antagonist, e.g., diphenhydramine
- Intravenous corticosteroid agent with a short biological half-life, e.g., methylprednisolone

Compliance with this requirement was confirmed for all 27 subjects. No dose escalation was permitted above the MTD identified by the DMC. However, at the investigator's discretion, the dose for any subject in this expansion cohort could be reduced by 25%–33% based upon a subject's toxicity at the MTD (Supplementary Fig. S1).

Patients could receive up to six total cycles in absence of PD. A separate dosing extension protocol was provided to all sites participating in this study that allowed additional dosing if the patient continued to respond to treatment.

In the dose escalation study, 6 cohorts received MT-3724 at 5 (*n* = 3), 10 (*n* = 3), 20 (*n* = 3), 50 (*n* = 4), 100 (*n* = 2), and 75 (*n* = 6) μg/kg/dose, respectively. Cohort 7 (*n* = 6) was treated at the MTD (75 μg/kg/dose), which was defined as the dose level below the maximum administered dose (MAD) where 1 or fewer patients experienced DLT. MAD was defined as the dose level where two or more patients experienced DLT.

### Patients

Adult patients with CD20-positive r/rNHL who had exhausted all standard of care options for lymphoma, including ≥1 anti-CD20–containing regimen, were eligible. During conduct of the study, after the initial 3+3 dose evaluation, it became clear that circulating anti-CD20 mAbs such as rituximab may preclude efficacy, prompting a protocol amendment to require both an anti-CD20 mAb washout period of ≥5 half-lives and a serum rituximab level below the limit of detection (<500 ng/mL) for patients exposed within one year of screening. Additional eligibility criteria are shown in Supplementary Table S1.

Subgroup analyses were conducted according to histology; patients were considered transformed if they had an original disease histology of follicular lymphoma (FL) and subsequently had DLBCL histology prior to screening. Composite histology was defined as the presence of two or more morphologically and immunophenotypically distinct types of lymphoma involving the same anatomic site or tissue at the time of diagnostic biopsy.

### Pharmacokinetic Analyses

In C1, blood samples were collected on D1, 5, 8, 12, and once between D23–25; in C2–5, blood draws were taken on D1 and once between D3–18 (see also Supplementary Table S2).

MT-3724 serum concentrations were determined using electrochemiluminescence with the Meso Scale Discovery (MSD) platform. The pharmacokinetic analysis set included patients who received MT-3724 and had sufficient serum concentration data to determine primary pharmacokinetic parameters.

### Pharmacodynamic Analyses

During dose escalation and expansion studies, whole blood samples for pharmacodynamic, CD19^+^ B-cell count, were collected at screening, C1D8, and C1D23 (dose escalation only). After C1, pharmacodynamic samples were collected predose on D1 of treatment (odd cycles only for dose escalation) and at the end-of-treatment (EOT) visit

### Safety

Adverse events (AE) were classified using MedDRA v17.0. Toxicity was graded using the Common Toxicity Criteria guidelines from the National Cancer Institute (CTCAE v. 4.03). The safety analysis set included patients who received at least one dose of MT-3724.

### Disease Assessment and Response Criteria

Disease response was evaluated radiologically following completion of even-numbered cycles of MT-3724 and a final safety assessment following termination of MT-3724. Tumor response was evaluated by the investigator according to the IWG Response Criteria for Clinical Trials (13). Exploratory efficacy analyses [objective response rate (ORR)] were conducted in a subset of patients with mixed or transformed DLBCL histology who had ≥1 tumor reevaluation.

### Immunogenicity

Antidrug antibody (ADA) and neutralizing antibody (Nab) assessments were conducted at screening, D23, predose for C2–5, and EOT. Additional ADA samples were taken if clinically indicated. Evaluable patients had ≥1 sample during treatment, excluding screening. The presence of ADAs and Nabs were assessed with the MSD platform by bridging electrochemiluminescence and competitive ligand binding methods, respectively. Treatment-induced ADA patients were defined as those who were ADA negative at screening but became ADA positive after treatment; treatment-boosted patients were ADA positive at screening and maintained increased ADA titers following treatment.

### Data Availability Statement

The authors certify that this manuscript reports original clinical trial data. Data reported in this manuscript are available within the article or posted publicly at www.clinicaltrials.gov, according to the required timelines. Additional data from the study are available upon request from the corresponding author.

## Results

### Patients

Overall, 27 patients were enrolled (Table 1; Supplementary Fig. S2). Histologies included DLBCL (*n* = 17, 63.0%), FL (*n* = 6, 22.2%), composite DLBCL/FL (*n* = 2, 7.4%), and MCL (*n* = 2, 7.4%). Five patients completed all planned therapy. Overall, 14 (51.9%) discontinued due to PD, 5 (18.5%) due to AEs, and 1 each (3.7%) due to patient request, physician decision, and death (Supplementary Table S3). Patients were predominantly female (63%), white (85.2%), and non-Hispanic (88.9%), with a median age of 66 years (range, 34–78). The median number of prior lines of therapy for NHL was 4, ranging from 1 to 11. The majority of patients (10 of 18) who screen failed did so because of measurable rituximab levels at screening following the protocol amendment stipulating that patients’ rituximab levels had to be below the lower limit of detection of the rituximab assay.

**TABLE 1:** Demographics and Baseline Characteristics

Parameters	5 μg/kg/dose *n* = 3	10 μg/kg/dose *n* = 3	20 μg/kg/dose *n* = 3	50 μg/kg/dose *n* = 4	100 μg/kg/dose *n* = 2	75 μg/kg/dose *n* = 6	MTD Expansion cohort^a^ *n* = 6	Overall *n* = 27
Gender, *n* (%)								
Male	3 (100.0)	1 (33.3)	1 (33.3)	1 (25.0)	1 (50.0)	2 (33.3)	1 (16.7)	10 (37.0)
Race, *n* (%)								
White	2 (66.7)	3 (100.0)	3 (100.0)	2 (50.0)	2 (100.0)	5 (83.3)	6 (100.0)	23 (85.2)
Black	0	0	0	0	0	0	0	0
Asian	1 (33.3)	0	0	0	0	1 (16.7)	0	2 (7.4)
Other	0	0	0	2 (50.0)	0	0	0	0
Ethnicity								
Hispanic	0	0	0	2 (50.0)	0	0	0	2 (7.4)
Non-Hispanic	3 (100.0)	3 (100.0)	3 (100.0)	2 (50.0)	2 (100.0)	6 (100.0)	5 (83.3)	24 (88.9)
Unknown	0	0	0	0	0	0	1 (16.7)	1 (3.7)
Median age, years^b^ (range)	76 (66, 78)	73 (61, 78)	65 (50, 78)	62 (34, 73)	65 (62, 68)	70 (61, 70)	58 (44, 72)	66 (34–78)
Median height, cm (range)	164 (158, 174)	164 (161, 169)	171 (164, 182)	157 (150, 165)	168 (166, 170)	163 (155, 173)	161 (152, 186)	163 (150–186)
Median weight, kg (range)	66 (63, 95)	78 (69, 97)	63 (56, 78)	70 (63, 79)	97 (85, 109)	69 (51, 109)	97 (64, 154)	78 (51–154)
Baseline ECOG PS, *n* (%)								
0	2 (66.7)	1 (33.3)	1 (33.3)	0	1 (50.0)	1 (16.7)	4 (66.7)	10 (37.0)
1	1 (33.3)	2 (66.7)	1 (33.3)	2 (50.0)	1 (50.0)	5 (83.3)	1 (16.7)	13 (48.1)
2	0	0	1 (33.3)	2 (50.0)	0	0	1 (16.7)	4 (14.8)
NHL type, *n* (%)								
DLBCL	2 (66.7)	1 (33.3)	3 (100.0)	2 (50.0)	2 (100.0)	3 (50.0)	4 (66.7)	17 (63.0)
Mixed DLBCL	0	0	0	0	0	0	2 (33.3)	2 (7.4)
FL	1 (33.3)	2 (66.7)	0	1 (25.0)	0	2 (33.3)	0	6 (22.2)
MCL	0	0	0	1 (25.0)	0	1 (16.7)	0	2 (7.4)
Screening RTX level in serum, *n* (%)								
Positive (≥500 ng/mL^c^ in serum)	1 (33.3)	0	2 (66.7)	3 (75.0)	0	2 (33.3)	0	8 (29.6)
Negative (<500 ng/mL in serum)	2 (66.7)	3 (100.0)	1 (33.3)	1 (25.0)	2 (100.0)	4 (66.7)	6 (100.0)	19 (70.4)

Abbreviations: DLBCL, diffuse large B-cell lymphoma; ECOG PS, Eastern Cooperative Oncology Group Performance Status; FL, follicular lymphoma; MCL, mantle cell lymphoma; NHL, non-Hodgkin's lymphoma; RTX, rituximab.

^a^50 or 75 μg/kg/dose in the respective patients treated in the dose expansion study.

^b^Including anti-CD20 therapy.

^c^Limit of detection.

### MTD

The first 2 subjects treated at the 100 μg/kg/dose had 1 DLT each (CTC grade 3 pneumonia and CTC grade 2 ileus). In addition, 1 of these subjects had grade 3 muscle weakness related to treatment and multiple AEs indicative of systemic inflammatory response in the first cycle of treatment. Although the constellation of symptoms in the 2 subjects treated with the 100 μg/kg/dose MT 3724 differed in some ways, they both also had a drop in serum albumin. In one subject, estimated glomerular filtration rate decreased from 85 mL/min/1.73 m^2^ to 52 mL/min/1.73 m^2^), serum albumin decreased from 4.2 g/dL to 2.0 g/dL, and the other subject experiencing grade 2 ileus had neutrophilia of up to 2.7 × 10^10^/L. The DMC determined that 100 μg/kg/dose was the MAD, an interim lower dose cohort at 75 μg/kg/dose was added.

Per protocol definition, the MTD was initially defined as 75 μg/kg/dose. However, two (both with high body weights, 154 and 97 kg, respectively) of the first three patients treated at 75 μg/kg in the dose expansion study had grade 2 capillary leak syndrome (CLS) considered related to MT-3724, although not meeting DLT criteria. The third patient had a serious AE (SAE) of grade 3 edema. Thus, the MTD was reduced to 50 μg/kg/dose, with a 6,000 μg/dose cap instituted at the recommendation of the Data and Safety Monitoring Board.

### Pharmacokinetics and Pharmacodynamics

**FIGURE 1:**
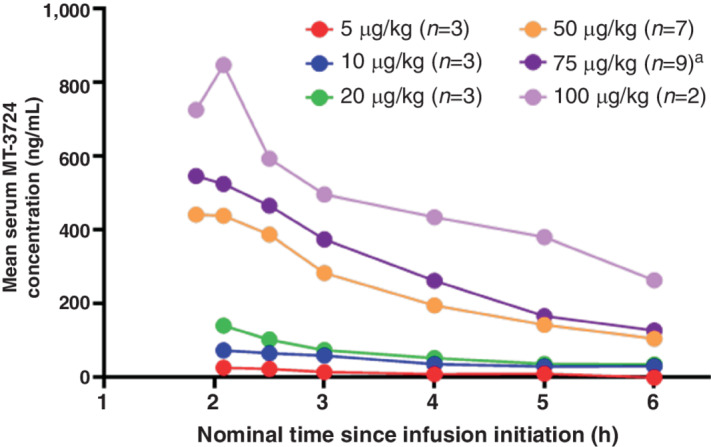
Mean serum MT-3724 concentration versus time plot with dose overlaid for cycle 1 (linear). Nominal time is relative to the start of infusion; predose concentrations were collected for the 50, 75, and 100 μg/kg dose groups, were all 0, and are not displayed in this figure. ^a^Hour 5, *n* = 8; hour 6, *n* = 7.

The pharmacokinetic population consisted of 27 patients. On C1D1, serum concentrations increased dose-proportionately from 5 to 100 μg/kg (Fig. 1); peak concentrations occurred 10 minutes prior to end of infusion or 5 minutes post-end of infusion. C1D1 *C*_max_ and area under the curve (AUC) values increased with dose level but were variable, with geometric coefficient of variation (where calculable) ranging from 42.7% to 77.8% for *C*_max_ and 35.1%–95.2% for AUC_0–4_. *T*_max_ was similar across dose levels, with median values ranging from 1.85 to 3.29 hours post-start of infusion. The t_1∕2_ values were not reportable for the 5 and 10 μg/kg cohorts, but geometric mean values were similar for the 20–100 μg/kg cohorts, ranging from 1.50 to 2.78 hours (Supplementary Table S4). At the sixth MT-3724 dose on C1D12, the geometric mean accumulation ratio was 0.72 for 37.5 μg/kg, 1.29 for 50 μg/kg, and 1.49 for 75 μg/kg (Supplementary Table S5).

**FIGURE 2:**
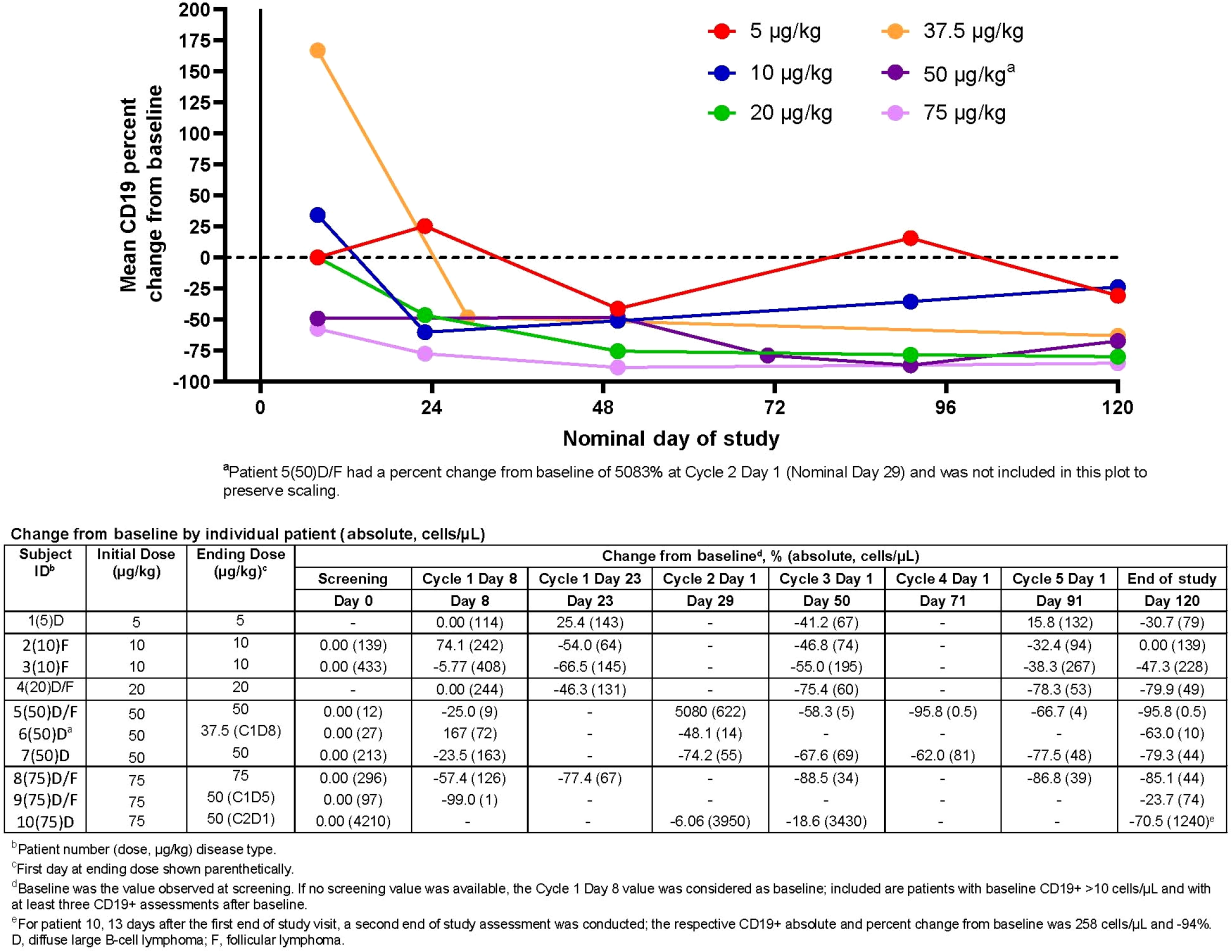
Overlay of mean CD19^+^ percent change from baseline versus time by actual dose.

Circulating CD19^+^ B-cells decreased after one cycle of treatment and remained decreased through the end of study (EOS; *n* = 10; Fig. 2). On C1D23 and C2D1, 7 of 9 patients demonstrated decreased percentage of CD19^+^ cells compared with baseline. At EOS, all treatment arms had a net decrease in CD19-expressing cells compared to baseline [5 μg/kg, −30.7% (*n* = 1); 10 μg/kg, −23.7% (*n* = 2); 20 μg/kg, −79.9% (*n* = 1); 37.5 μg/kg, −63.0% (*n* = 1); 50 μg/kg, −67.3% (*n* = 4); Fig. 2]. Generally, increased dose levels were associated with a lower nadir in CD19^+^ B cells; decreases in CD19^+^ cell counts were also observed in patients who were Nab-positive (Supplementary Fig. S3).

### Safety

In the safety population (*n* = 27), median duration of exposure was 39 days (range, 5–362) and median number of doses was 11 (range, 2–66).The most common treatment-emergent AEs (TEAE) of any grade or causality were peripheral edema (*n* = 17, 63%), diarrhea (*n* = 11, 40.7%), fatigue (*n* = 11, 40.7%), and myalgia (*n* = 11, 40.7%). Overall, 26 patients (96.3%) had ≥1 treatment-related TEAE (TR-TEAE; Supplementary Table S3); 13 patients (48.1%) had ≥1 TR-TEAE of ≥Grade 3 severity. The most common TR-TEAEs ≥Grade 3 severity included myalgia (*n* = 3, 11.1%), neutropenia, pneumonia, decreased lymphocyte count, and arthralgia (each *n* = 2, 7.4%; Table 2). Fourteen patients (51.9%) had 27 unique SAEs (Supplementary Table S3). The most common SAEs were pneumonia (*n* = 4, 14.8%), acute renal failure (*n* = 3, 11.1%), and thrombocytopenia and hypercalcemia (*n* = 2, 7.4%). Nine SAEs in 6 patients were treatment-related (Supplementary Table S3), and included pneumonia (*n* = 2), edema, peripheral edema, ileus, viral infection, hypertension, acute renal failure, and muscular weakness (each *n* = 1). Five of these six patients received doses above the MTD of 50 μg/kg.

**TABLE 2:** Summary of treatment-related ≥grade 3 TEAEs by worst CTCAE grade (safety set)^a^

Preferred term, *n* (%)	5 μg/kg/dose (*n* = 3)	10 μg/kg/dose (*n* = 3)	20 μg/kg/dose (*n* = 3)	50 μg/kg/dose (*n* = 4)	100 μg/kg/dose (*n* = 2)	75 μg/kg/dose (*n* = 6)	MTD Expansion cohort^b^ (*n* = 6)	Overall (*N* = 27)
At least 1 grade 3 treatment-related TEAE	0	0	0	1 (25.0)	2 (100.0)	1 (16.7)	6 (100.0)	10 (37.0)
Acute renal failure	0	0	0	0	0	1 (16.7)^c^	0	1 (3.7)^c^
Arthralgia	0	0	0	0	0	0	2 (33.3)	2 (7.4)
Edema	0	0	0	0	0	0	1 (16.7)	1 (3.7)
Fatigue	0	0	0	0	0	0	1 (16.7)	1 (3.7)
Headache	0	0	0	0	0	0	1 (16.7)	1 (3.7)
Hypertension	0	0	0	1 (25.0)	0	0	0	1 (3.7)
Hypokalemia	0	0	0	0	0	0	1 (16.7)	1 (3.7)
Leukopenia	1 (33.3)^c^	0	0	0	0	0	0	1 (3.7)^c^
Lymphocyte count decreased	0	0	0	1 (25.0)	0	0	1 (16.7)	2 (7.4)
Muscular weakness	0	0	0	0	1 (50.0)	0	0	1 (3.7)
Myalgia	0	0	0	0	1 (50.0)	0	2 (33.3)	3 (11.1)
Neutropenia	1 (33.3)^c^	0	0	1 (25.0)^c^	0	0	0	2 (7.4)^c^
Neutrophil count decreased	0	0	0	0	0	0	1 (16.7)	1 (3.7)
Oropharyngeal candidiasis	0	0	0	0	0	1 (16.7)	0	1 (3.7)
Pneumonia	0	0	0	0	1 (50.0)	0	1 (16.7)	2 (7.4)
Rash	0	0	0	0	0	0	1 (16.7)	1 (3.7)
Upper respiratory tract infection	0	0	0	0	0	1 (16.7)	0	1 (3.7)

Abbreviations: CTCAE, Common Terminology Criteria for Adverse Events (version 4.03), PT, preferred term; SOC, system organ class.

^a^If a patient experienced more than one event in a given SOC, that patient is counted once for that SOC. If a patient experienced more than one event with a given PT, that patient is counted only once for that PT.

^b^50 or 75 μg/kg/dose in the respective patients treated in the dose expansion study.

^c^Grade 4 treatment-related TEAE; all other treatment-related TEAEs were grade 3.

An adverse event of special interest (AESI) was capillary leak syndrome (CLS), which is known to be associated with other immunotoxins. CLS includes clinical signs and symptoms such as hypotension, edema, weight gain, and hypoalbuminemia. Since mild–moderate grades of CLS may not be always reported as such, a retrospective analysis was conducted by evaluating patients with AEs that were indicative of possible CLS. Patients with hypotension, hypoalbuminemia (reported or lab result <3g/dL), and edema/peripheral edema or weight gain that occurred in the same cycle were included. Patients with ≥2 events were considered to have possible CLS.

Two patients in Cohort 7 (75 μg/kg/dose) were diagnosed with grade 2 CLS that was considered related to MT-3724, and 10 additional patients had symptoms suggestive of CLS. Among 12 patients with diagnosed or suspected CLS, the most common events were edema (*n* = 10), hypoalbuminemia (*n* = 10), and hypotension (*n* = 5), with a most severe grade of 2 (*n* = 9; 75%).

CLS signs and symptoms tended to improve to grade 1 or resolve after drug interruption. A dose response appeared present, as the symptoms increased in severity at higher dose levels, especially in patients with obesity who had received a high total dose.

### Efficacy

**FIGURE 3:**
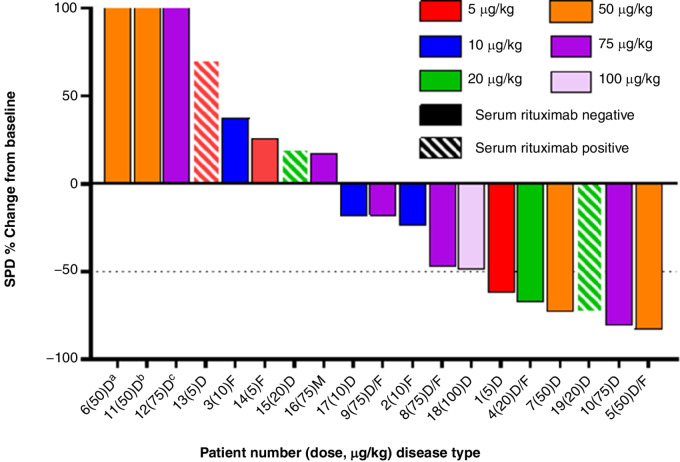
Waterfall plot of largest percentage change in SPD by patients with DLBCL or mixed FL/DLBCL histology and treatment group (FAS). ^a^SPD, 303.9%. ^b^SPD, 201.4%. ^c^SPD, 182.5%. Dotted line indicated −50% change in SPD, meeting requirement for PR. D, diffuse large B-cell lymphoma; F, follicular lymphoma; FAS, full analysis set; M, mantle cell lymphoma; PR, partial response; SPD, sum of product diameters.

**FIGURE 4:**
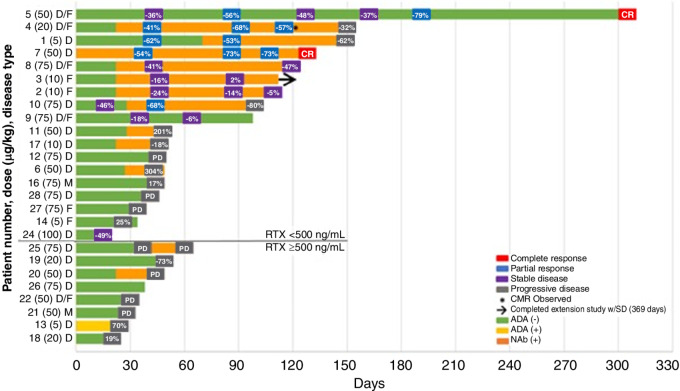
Swimmer plot with ADA/Nab status. ADA, antidrug antibody; CMR, complete metabolic response; D, diffuse large B-cell lymphoma; F, follicular lymphoma; M, mantle cell lymphoma; Nab, neutralizing antibody; RTX, rituximab; SD, stable disease.

Among the 25 patients evaluable for efficacy, the ORR was 21.7%; two patients (8.7%) had a complete response (CR), three patients (13.0%) had partial response (PR), and 5 patients (21.7%) had stable disease (SD; Supplementary Table S6). Among the 5 patients with SD were two patients who had a decrease in tumor burden of 48%-49% that did not meet criteria for PR. The percent change from baseline in the sum product of perpendicular diameters (SPD) for target tumor lesion size is shown in Fig. 3. Length of time on study and tumor response are summarized in Fig. 4 for the 19 subjects with unmeasurable rituximab levels at screening.

Seventeen patients were serum rituximab-negative at screening [(DLBCL including composite histology), (*n* = 12); non-DLBCL, *n* = 5)]. Among these, median PFS was 100.0 days (95% CI, 45.0-not evaluable). Among serum rituximab negative patients with DLBCL, the ORR was 41.7% [5/12 patients: 95% confidence interval (CI), 15.2%–72.3%; in a previous analysis, one patient's histologic classification was initially reported as mixed FL/DLBCL but was later changed to FL. In that analysis, the ORR was reported to be 38.5%; the patient had SD as best response.]; with a median duration on study of 130 days (range: 85–288).

Among the 5 serum-rituximab negative patients with non-DLBCL, there were no responders; however, one patient (initially diagnosed with DLBCL, who subsequently relapsed with FL following autologous SCT) had SD, with a 47% tumor reduction.

### Immunogenicity

Among 27 patients screened for ADA, 18.5% (5/27) had nascent ADAs at screening; 25 patients were evaluable beyond screening. Of these, 10 (40.0%) were confirmed negative and 15 were confirmed positive (60.0%) by EOS. Among the ADA-positive patients, 12 (80.0%) were treatment-induced and 3 (20.0%) were treatment-boosted. The proportion of patients positive for ADAs was 18.5% (5/27) at screening and 75.0% (6/8) patients at C3D1 (Supplementary Table S7).

Among the 19 ADA-positive samples, all but two were Nab-positive. At screening, 5 of 27 (18.5%) ADA-positive samples were Nab-positive, rising to 13 of 20 (65.0%) at EOS. Two patients were initially Nab-negative then Nab-positive by the end of assessment. Four out of 5 patients who had a best response of CR or PR were ADA positive, and 3 of 5 patients with a best response of stable disease, also tested ADA positive (Fig. 4). There was no apparent association between adverse events and ADA positivity.

## Discussion

MT-3724 is an ETB with a unique mechanism of action, capable of binding to CD20 and forcing internalization via SLT-A and thus inducing potent cell death**,** despite CD20 being known as a generally noninternalizing receptor (15). In patients with r/rNHL, anti-CD20 treatments have been limited to mAbs and radioimmunotherapeutics, as antibody–drug conjugates against CD20 are not effective due to the lack of internalization. Considering the potential ability of MT-3724 to evade mechanisms of resistance commonly observed with other anti-CD20 mAbs, it was important to evaluate the efficacy and safety of MT-3724 in patients with r/rNHL (16).

The results demonstrate that MT-3724 is tolerable when administered at the MTD (50 μg/kg/dose with a maximum dose of 6,000 μg/dose) and active in a subset of patients with r/rNHL who have no measurable rituximab levels before starting treatment with MT-3724: in this subset the ORR of this single-agent therapy was 41.7%.

CLS was evident or suspected at 75 μg/kg/dose and appears manageable as patients recovered and tolerated continued treatment with MT-3724 at 50 μg/kg. Two patients treated at 75 μg/kg with confirmed grade 2 CLS appeared to be correlated with body weight and the resultant higher total doses of 11,572.5 μg/infusion and 7,207.5 μg/infusion. There were no signs of recurrence with subsequent doses, possibly due to premedication with corticosteroids, antihistamines, and acetaminophen and prompt administration of albumin, fluid replacements, and diuretics as needed. While rare, CLS has been observed in response to a variety of drugs, including immunotoxins, immunomodulators, and other antineoplastics (17). Other immunotoxins containing an intact or truncated toxin have demonstrated > grade 3 CLS in preclinical and clinical studies (18), incurring a “black box” warning for the three FDA-approved immunotoxins (denileukin difitox, moxetumomab pasudotox-tdfk, and tagraxofusp-erzs) and suggesting a class effect (19–21).

Pharmacokinetic analysis indicated that *C*_max_ and AUC values increased in an approximately dose-dependent manner. *T*_max_ values were generally observed at the first sample time after end of infusion and were similar across dose levels, with medians ranging from 1.85 to 3.29 hours post-start of infusion. This was largely due to variability in infusion duration, which was decreased from 2 hours to 1 hour between protocol versions. Little to no accumulation was observed by the sixth MT-3724 dose on C1D12. Due to highly variable data, small sample size, short sampling period, and inconsistent sampling, pharmacokinetics/pharmacodynamics relationships were difficult to discern. Nevertheless, MT-3724 was associated with decreased CD19^+^ B-cell counts relative to baseline in a dose-dependent manner, with a decrease of −23.7% (10 μg/kg) up to −95.8% (50 μg/kg). This effect was sustained throughout the study despite emergence of ADAs and Nabs, suggesting these antibodies may not abrogate the ability of MT-3724 to bind and kill CD20-expressing cells.

ADAs were observed at all dose levels and almost all ADA-positive patients were also Nab-positive. While the proportion of ADA-positive patients increased over the course of treatment (Supplementary Table S6); elevated ADA/Nabs interestingly did not appear to correlate with safety events, tumor response, or clinical outcomes.

This study had several limitations. First, the open-label design could bias the assessment of adverse events. However, the use of objective, laboratory assessments for CLS reduce this limitation. Second, the small number of patients in each study limits the power to detect differences between groups with regards to efficacy. In addition, histologic characterization and tumor CD20 expression relied on historical documentation rather than contemporaneous biopsies and centralized pathology, and efficacy analyses in all or subsets of patients were conducted *post hoc* in this exploratory, first-in-human trial.

In conclusion, this first-in-human, phase Ia/b, study of MT-3724 in patients with r/r NHL determined an MTD of 50 μg/kg/dose with a maximum dose of 6,000 μg/dose. All patients who responded to treatment had DLBCL or mixed composite DLBCL/FL histology (Fig. 4). The unique mechanism of action of MT-3724 which forces internalization against CD20, a noninternalizing receptor, once bound to the target and causes cytotoxicity by the enzymatic and permanent inactivation of ribosomes, coupled with its relatively tolerable safety profile, make it a potentially suitable therapy for patients with r/rDLBCL either as a single agent or in combination and in the current era may provide an additional treatment strategy for patients who failed to respond to CAR T-cell therapy.

## Supplementary Material

Supplementary Tables 1-7, Figures 1-3 Supplementary Table 1. Additional Study Eligibility Criteria. Supplementary Table 2. Pharmacokinetic Serum Sample Collection. Supplementary Table 3. Summary of Adverse Events and Discontinuations. Supplementary Table 4. Summary of Serum MT-3724 PK Parameters by Treatment on Cycle 1 Day 1. Supplementary Table 5. Summary of Serum MT-3724 PK Parameters by Treatment on Cycle 1 Day 12. Supplementary Table 6. Best Overall Response, Objective Response Rate, and Disease Control Rate (FAS). Supplementary Table 7. Summary of ADA Incidence by Actual Dose. Supplementary Figure 1. Study Design for Dose Escalation and Dose Expansion. Supplementary Figure 2. CONSORT diagram. Supplementary Figure 3. Individual CD19+ Nadir Percent Change from Baseline.
