# Supplementary material for: Safety and Efficacy of Engineered Toxin Body MT-3724 in Relapsed or Refractory B-cell Non-Hodgkin's Lymphomas and Diffuse Large B-cell Lymphoma
Source: Cancer Res Commun. 2022 May 5;2(5):307–15. doi: 10.1158/2767-9764.CRC-22-0056 (PMC9981212; doi:10.1158/2767-9764.CRC-22-0056)
Supplement: Supplementary Tables 1-7, Figures 1-3 — Supplementary Table 1. Additional Study Eligibility Criteria. Supplementary Table 2. Pharmacokinetic Serum Sample Collection. Supplementary Table 3. Summary of Adverse Events and Discontinuations. Supplementary Table 4. Summary of Serum MT-3724 PK Parameters by Treatment on Cycle 1 Day 1. Supplementary Table 5. Summary of Serum MT-3724 PK Parameters by Treatment on Cycle 1 Day 12. Supplementary Table 6. Best Overall Response, Objective Response Rate, and Disease Control Rate (FAS). Supplementary Table 7. Summary of ADA Incidence by Actual Dose. Supplementary Figure 1. Study Design for Dose Escalation and Dose Expansion. Supplementary Figure 2. CONSORT diagram. Supplementary Figure 3. Individual CD19+ Nadir Percent Change from Baseline. [file crc-22-0056-s01.docx]

**Supplementary Tables and Figures**

**Supplementary Table 1. Additional Study Eligibility Criteria**

| Eligibility Criteria | Exclusion Criteria |
| --- | --- |
| - Males or females aged 18 years or older | - NHL diagnosis confirmed by histology based only on bone marrow biopsies and/or fine needle aspirates was not accepted |
| - Patients had an Eastern Cooperative Oncology Group Performance Status ≤2 | - History of cancer other than basal cell carcinoma or cervical intraepithelial neoplasia, unless the previous cancer was treated and the patient remained disease free for ≥5 years prior to screening, or if their physician considered them to be at <30% risk of relapse |
| - Patients must have received all approved therapies known to provide clinical benefit for their disease subtype. For patients with lymphomas for which high-dose chemotherapy and autologous stem cell transplant (HD-ASCT) was considered standard curative therapy, patients must have been ineligible for HD-ASCT, experienced relapse following HD-ASCT, or refused HD-ASCT. | - Significant infection within 2 weeks of first dose |
| - Previously confirmed immunohistological diagnosis with a characteristic CD20+ B-cell immunophenotype according to the World Health Organization (WHO) criteria was acceptable, but reconfirmed at discretion of the investigators | - Systemic corticosteroid therapy (>20 mg/day prednisone equivalent dose) within 2 weeks of first dose |
| - Confirmed B-cell clonality at screening | - Recipients of allogeneic hematopoietic stem cell transplantation |
| - Laboratory requirements:   - Absolute neutrophil count >1,000/uL   - Platelet count >50,000/uL^a^   - Total bilirubin < 1.5 x ULN   - Aspartate aminotransferase and alanine aminotransferase ≤2.5 x ULN, or ≤5 x ULN if liver metastases were present   - Creatinine clearance ≥ 60 mL/min |  |

^a^Platelet count ≥ abno/µL was allowed if (i) the patient was free of any clinically significant signs/symptoms of bleeding, (ii) the thrombocytopenia was secondary to bone marrow infiltration by disease, and (iii) the thrombocytopenia was not secondary to increased platelet consumption or the result of previous marrow suppressing treatment or infection.

ULN, upper limit of normal.

This table is an additional detailed list of the patient requirements for eligibility in the study. This includes the demographic, medical history, and specific disease requirements to be enrolled in the study, as well any prior treatments or disease characteristics that would preclude inclusion in the study.

**Supplementary Table 2. Pharmacokinetic Serum Sample Collection**

|  | **Pre-infusion^a^** | **10 min prior to end**  **of primary infusion^b^** | **0.083 hr**  **(± 1 min)**  **Post-EOI** | **0.5 hr  Post-EOI** | **1 hr Post-EOI** | **2 hr  (± 5 min)  Post-EOI** | **3 hr  Post-EOI** | **4 hr  (± 10 min)  Post-EOI** | **Concurrent with immunogenicity draws** |
| --- | --- | --- | --- | --- | --- | --- | --- | --- | --- |
| **Cycle 1** | | | | | | | | | |
| Day 1 | **X** | **X** | **X** | **X** | **X** | **X** | **X** | **X** |  |
| Day 5 |  | **X** | **X** |  |  | **X** |  |  |  |
| Day 8 | **X** |  |  |  |  |  |  |  |  |
| Day 12 | **X** | **X** | **X** | **X** | **X** | **X** |  |  |  |
| Days 23-25^c^ |  |  |  |  |  |  |  |  | **X** |
| **Cycles 2-5** | | | | | | | | | |
| Day 1 |  |  | **X** |  |  |  |  |  |  |

^a^Days 3-18: Pre-infusion sample collected within 4 hours prior to the start of primary infusion.

^b^Cohorts 50 µg/kg and above only.

^c^Dose escalation only.

EOI, end of infusion.

This table shows the specific days and timepoints at which pharmacokinetic serum samples were collected during Cycles 1 and 2-5.

**Supplementary Table 3. Summary of Adverse Events and Discontinuations**

|  | **5 µg/kg/dose**  **(n=3)** | **10 µg/kg/ dose**  **(n=3)** | **20 µg/kg/ dose**  **(n=3)** | **50 µg/kg/ dose**  **(n=4)** | **100 µg/kg/ dose**  **(n=2)** | **75 µg/kg/ dose**  **(n=6)** | **MTD Expansion Cohort**  **(n=6)^a^** | **Overall**  **(N=27)** |
| --- | --- | --- | --- | --- | --- | --- | --- | --- |
| Patients with ≥1 TEAE, n (%) | 3 (100.0) | 3 (100.0) | 3 (100.0) | 4 (100.0) | 2 (100.0) | 6 (100.0) | 6 (100.0) | 27 (100.0) |
| Patients with ≥1 treatment-related TEAEs, n (%) | 3 (100.0) | 3 (100.0) | 2 (66.7) | 4 (100.0) | 2 (100.0) | 6 (100.0) | 6 (100.0) | 26 (96.3) |
| Patients with ≥1 SAEs, n (%) | 1 (33.3) | 0 | 2 (66.7) | 4 (100.0) | 2 (100.0) | 3 (50.0) | 2 (33.3) | 14 (51.9) |
| Patients with ≥1 treatment-related SAE, n (%) | 0 | 0 | 0 | 1 (25.0) | 2 (100.0) | 2 (33.3) | 1 (16.7) | 6 (22.2) |
| Patients with ≥1 DLT, n (%) | 0 | 0 | 0 | 0 | 2 (100.0) | 0 | 0 | 2 (7.4) |
| Patients with ≥1 treatment-related TEAE leading to death, n (%) | 0 | 0 | 0 | 0 | 0 | 0 | 0 | 0 |
| **Early discontinuation, n (%)^b^** | **2 (66.7)** | **1 (33.3)** | **3 (100.0)** | **4 (100.0)** | **2 (100.0)** | **5 (83.3)** | **5 (83.3)** | **22 (81.5)** |
| Disease progression | 2 (66.7) | 1 (33.3) | 3 (100.0) | 1 (25.0) | 0 | 4 (66.7) | 3 (50.0) | 14 (51.9) |
| Adverse events | 0 | 0 | 0 | 2 (50.0) | 2 (100.0) | 1 (16.7) | 0 | 5 (18.5) |
| Patient request | 0 | 0 | 0 | 0 | 0 | 0 | 1 (16.7) | 1 (3.7) |
| Lost to follow-up | 0 | 0 | 0 | 0 | 0 | 0 | 0 | 0 |
| Physician decision | 0 | 0 | 0 | 0 | 0 | 0 | 1 (16.7) | 1 (3.7) |
| Death | 0 | 0 | 0 | 1 (25.0) | 0 | 0 | 0 | 1 (3.7) |
| ^a^50 or 75 µg/kg/dose in the respective patients treated in the dose expansion study.  ^b^Study discontinuation is based on whether patients completed the number of planned cycles based on the protocol version in which they enrolled.  DLT, dose-limiting toxicity; MTD, maximum tolerated dose; SAE, serious treatment-emergent adverse event; TEAE, treatment-emergent adverse event. | | | | | | | | |

This table provides additional details of the adverse events and discontinuations that occurred on study. Data are presented by dose level and as a total for the overall study.

**Supplementary Table 4. Summary of Serum MT-3724 PK Parameters by Treatment on Cycle 1 Day 1^a^**

| **Treatment** | **Statistic** | **C_max_** | **T_max_** | **T_last_** | **AUC_last_** | **AUC_0-4_** | **AUC_inf_** | **t_½_** |
| --- | --- | --- | --- | --- | --- | --- | --- | --- |
|  |  | **(ng/mL)** | **(h)** | **(h)** | **(h*ng/mL)** | **(h*ng/mL)** | **(h*ng/mL)** | **(h)** |
| 5 µg/kg | **N** | 1 | 1 | 1 | 1 | 1 | 0 | 0 |
|  | **Geometric Mean** | 57.5 | NC | NC | 170 | 140 | NC | NC |
|  | **Geometric CV%** | NC | NC | NC | NC | NC | NC | NC |
|  | **Min** | 57.5 | 2.10 | 5.00 | 170 | 140 | NC | NC |
|  | **Median** | 57.5 | 2.10 | 5.00 | 170 | 140 | NC | NC |
|  | **Max** | 57.5 | 2.10 | 5.00 | 170 | 140 | NC | NC |
| 10 µg/kg | **N** | 3 | 3 | 3 | 3 | 1 | 0 | 0 |
|  | **Geometric Mean** | 70.4 | NC | NC | 155 | 365 | NC | NC |
|  | **Geometric CV%** | 61.9 | NC | NC | 155 | NC | NC | NC |
|  | **Min** | 47.0 | 1.97 | 2.82 | 75.7 | 365 | NC | NC |
|  | **Median** | 55.0 | 2.08 | 3.00 | 88.8 | 365 | NC | NC |
|  | **Max** | 135 | 3.00 | 6.00 | 557 | 365 | NC | NC |
| 20 µg/kg | **N** | 3 | 3 | 3 | 3 | 3 | 3 | 3 |
|  | **Geometric Mean** | 132 | NC | NC | 333 | 278 | 451 | 2.07 |
|  | **Geometric CV(%)** | 46.6 | NC | NC | 73.2 | 56.2 | 87.5 | 58.6 |
|  | **Min** | 87.4 | 2.08 | 4.08 | 171 | 169 | 203 | 1.11 |
|  | **Median** | 124 | 2.08 | 5.92 | 342 | 264 | 497 | 2.73 |
|  | **Max** | 211 | 2.08 | 6.00 | 633 | 481 | 907 | 2.94 |
| 50 µg/kg | **N** | 7 | 7 | 7 | 7 | 7 | 7 | 7 |
|  | **Geometric Mean** | 445 | NC | NC | 1370 | 1040 | 1680 | 1.92 |
|  | **Geometric CV(%)** | 42.7 | NC | NC | 32.1 | 35.1 | 28.3 | 32.1 |
|  | **Min** | 211 | 2.08 | 5.92 | 775 | 576 | 1100 | 1.30 |
|  | **Median** | 473 | 2.32 | 6.00 | 1580 | 1130 | 1780 | 2.10 |
|  | **Max** | 644 | 2.58 | 6.82 | 1830 | 1440 | 2180 | 2.62 |
| 75 µg/kg | **N** | 9 | 9 | 9 | 9 | 9 | 9 | 9 |
|  | **Geometric Mean** | 486 | NC | NC | 1410 | 1060 | 1680 | 1.50 |
|  | **Geometric CV(%)** | 77.8 | NC | NC | 102 | 95.2 | 114 | 59.0 |
|  | **Min** | 134 | 1.70 | 3.00 | 207 | 217 | 220 | 0.392 |
|  | **Median** | 579 | 1.85 | 5.92 | 1680 | 1420 | 1890 | 1.66 |
|  | **Max** | 1220 | 4.42 | 8.00 | 2850 | 2840 | 3680 | 2.72 |
| 100 µg/kg | **N** | 2 | 2 | 2 | 2 | 2 | 2 | 2 |
|  | **Geometric Mean** | 828 | NC | NC | 2980 | 1650 | 3970 | 2.78 |
|  | **Geometric CV(%)** | NC | NC | NC | NC | NC | NC | NC |
|  | **Min** | 668 | 2.50 | 6.48 | 2030 | 1460 | 2550 | 2.30 |
|  | **Median** | 848 | 3.29 | 7.24 | 3200 | 1660 | 4360 | 2.83 |
|  | **Max** | 1030 | 4.08 | 8.00 | 4360 | 1870 | 6170 | 3.37 |

^a^Two patients in the pharmacokinetics population were excluded from analysis; one patient had fewer than three quantifiable post-dose concentrations and another patient had concentrations below the limit of quantification at all timepoints.

AUC_inf_, area under concentration-time curve from time 0 to infinity; AUC_last_, area under concentration-time curve from time 0 to the last quantifiable concentration; AUC_0-4_, area under concentration-time curve from time 0 to 4 hours; C_max_, maximum observed plasma/serum concentration; Geometric CV(%), Geometric percent coefficient of variation; Min, Minimum; Max, Maximum; NC, Not calculated; t_½_, half-life; T_last_, time to last measurable plasma concentration T_max_, time to maximum plasma concentration.

This table provides a summary of all pharmacokinetic parameters at Cycle 1 Day 1 for all patients in the pharmacokinetic population with evaluable data. Data are presented by dose level.

**Supplementary Table 5. Summary of Serum MT-3724 PK Parameters by Treatment on Cycle 1 Day 12**

| **Treatment** | **Statistic** | **C_max_** | **T_max_** | **T_last_** | **AUC_last_** | **AUC_0-4_** | **C_max_ / Dose AR** | **AUC_0-4_**  **Dose AR** |
| --- | --- | --- | --- | --- | --- | --- | --- | --- |
|  |  | **(ng/mL)** | **(h)** | **(h)** | **(h*ng/mL)** | **(h*ng/mL)** | **(RATIO)** | **(RATIO)** |
| 37.5 µg/kg | **N** | 1 | 1 | 1 | 1 | 1 | 1 | 1 |
|  | **Geometric Mean** | 352 | NC | NC | 782 | 782 | 0.767 | 0.724 |
|  | **Geometric CV%** | NC | NC | NC | NC | NC | NC | NC |
|  | **Min** | 352 | 2.08 | 4.00 | 782 | 782 | 0.767 | 0.724 |
|  | **Median** | 352 | 2.08 | 4.00 | 782 | 782 | 0.767 | 0.724 |
|  | **Max** | 352 | 2.08 | 4.00 | 782 | 782 | 0.767 | 0.724 |
| 50 µg/kg | **N** | 5 | 5 | 5 | 5 | 4 | 5 | 4 |
|  | **Geometric Mean** | 518 | NC | NC | 1250 | 1210 | 1.24 | 1.29 |
|  | **Geometric CV(%)** | 58.1 | NC | NC | 46.4 | 53.5 | 29.9 | 25.5 |
|  | **Min** | 202 | 1.85 | 3.92 | 582 | 582 | 0.895 | 1.01 |
|  | **Median** | 591 | 2.03 | 4.00 | 1480 | 1460 | 1.27 | 1.23 |
|  | **Max** | 782 | 2.08 | 4.47 | 1760 | 1760 | 1.81 | 1.81 |
| 75 µg/kg | **N** | 6 | 6 | 6 | 6 | 5 | 6 | 5 |
|  | **Geometric Mean** | 428 | NC | NC | 970 | 980 | 1.19 | 1.49 |
|  | **Geometric CV(%)** | 37.3 | NC | NC | 44.1 | 49.4 | 50.4 | 43.6 |
|  | **Min** | 262 | 1.83 | 3.90 | 459 | 457 | 0.550 | 0.850 |
|  | **Median** | 440 | 1.88 | 4.03 | 1040 | 1140 | 1.30 | 1.59 |
|  | **Max** | 682 | 2.13 | 4.08 | 1580 | 1570 | 1.96 | 2.28 |

**Patient 9(75)D/F** changed dose from 75 µg/kg to 50 µg/kg starting on Cycle 1 Day 5 (included in 50 µg/kg treatment group for PK analysis starting at Day 5); **Patient 6(50)D** changed dose from 50 µg/kg to 37.5 µg/kg starting on Cycle 1 Day 8 (included in 37.5 µg/kg treatment group for PK analysis starting at Day 8).

AR, accumulation ratio; AUC_last_, area under concentration-time curve from time 0 to the last quantifiable concentration; AUC_0-4_, area under concentration-time curve from time 0 to 4 hours; C_max_, maximum observed plasma/serum concentration; Geometric CV(%), Geometric percent coefficient of variation; Min, Minimum; Max, Maximum; NC, Not calculated; t_½_, half-life; T_last_, time to last measurable plasma concentration T_max_, time to maximum plasma concentration.

This table provides a summary of all pharmacokinetic parameters at Cycle 1 Day 12 for patients with evaluable data. Data are presented by dose level.

**Supplementary Table 6. Best Overall Response, Objective Response Rate, and Disease Control Rate (FAS)**

|  | **5 µg/kg /dose**  **(n=3)** | **10 µg/kg/dose**  **(n=3)** | **20 µg/kg/dose**  **(n=3)** | **50 µg/kg/dose**  **(n=4)** | **100 µg/kg/dose**  **(n=2)** | **75 µg/kg/dose**  **(n=6)** | **MTD Expansion Cohort^a^**  **(n=6)** | **Combined 50 µg/kg/**  **Dose^b^**  **(n=6)** | **Serum-RTX Negative**  **(n=17)** | **Serum-RTX Negative with DLBLC, including FL/DLBCL**  **(n=12_** | **Overall**  **(N=23)** |  |
| --- | --- | --- | --- | --- | --- | --- | --- | --- | --- | --- | --- | --- |
| BOR, n (%)^c^ |  |  |  |  |  |  |  |  |  |  |  |  |
| CR | 0 | 0 | 0 | 0 | 0 | 0 | 2 (33.3) | 2 (33.3) | 2 (11.8) | 2 (16.7) | 2 (8.7) |  |
| Unconfirmed/Uncertain CR | 0 | 0 | 0 | 0 | 0 | 0 | 0 | 0 | 0 | 0 | 0 |  |
| PR | 1 (33.3) | 0 | 1 (33.3) | 0 | 0 | 0 | 1 (16.7) | 0 | 3 (17.6) | 3 (25.0) | 3 (13.0) |  |
| SD | 0 | 2 (66.7) | 0 | 0 | 1 (100.0) | 1 (25.0) | 1 (16.7) | 0 | 5 (29.4) | 2 (16.7) | 5 (21.7) |  |
| PD | 2 (66.7) | 1 (33.3) | 2 (66.7) | 3 (100.0) | 0 | 3 (75.0) | 2 (33.3) | 4 (66.7) | 7 (41.2) | 5 (41.7) | 13 (56.5) |  |
| NE | 0 | 0 | 0 | 0 | 0 | 0 | 0 | 0 | 0 | 0 | 0 |  |
| ORR^d^, n (%), [95% CI] | 1 (33.3)  [0.8-90.6] | 0 | 1 (33.3)  [0.8-90.6] | 0 | 0 | 0 | 3 (50.0)  [11.8-88.2] | 2 (33.3)  [4.3-77.7] | 5 (29.4)  [10.3-56.0] | 5 (41.7)  [15.2-72.3] | 5 (21.7) [7.5-43.7] |  |
| ^a^50 or 75 µg/kg/dose in the respective patients treated in the expansion portion.  ^b^All patients with a starting dose of 50 µg/kg in the dose escalation or dose expansion portions.  ^c^Patients who progressed before the first post-baseline radiological assessment of tumor response are classified as PD, even though they did not undergo their first radiological tumor re-evaluation.  ^d^Proportion of patients with CR or PR.  BOR, best overall response; CR, complete response; DCR, disease control rate; DLBCL, diffuse large B-cell lymphoma; CI, confidence interval; FAS, full analysis set; FL, follicular lymphoma; MTD, maximum tolerated dose; NE, not evaluable; ORR, objective response rate; PD, progressive disease; PR, partial response; SD, stable disease. | | | | | | | | | | | | |

This table presents efficacy information, as summarized best overall response. Data are presented by dose level.

**Supplementary Table 7. Summary of ADA Incidence by Actual Dose**

| **Dose** | **Screen** | **C1D23** | **C2D1** | **C3D1** | **C4D1** | **C5D1** | **EOS** |
| --- | --- | --- | --- | --- | --- | --- | --- |
| **All Patients**  N  n (%) | 27  5 (18.5) | 15  6 (40.0) | 20  11 (55.0) | 8  6 (75.0) | 8  7 (87.5) | 7  6 (85.7) | 20  13 (65.0) |
| **5 µg/kg**  N  n (%) | 3  2 (66.6) | 1  0 | 1  0 | 1  0 | 1  1 (100.0) | 1  1 (100.0) | 3  2 (66.6) |
| **10 µg/kg**  N  n (%) | 3  0 | 3  3 (100.0) | 3  3 (100.0) | 2  2 (100.0) | 2  2 (100.0) | 2  2 (100.0) | 2  2 (100.0) |
| **20 µg/kg**  N  n (%) | 3  0 | 2  1 (50.0) | 2  1 (50.0) | 1  1 (100.0) | 1  1 (100.0) | 1  1 (100.0) | 2  1 (50.0) |
| **37.5 µg/kg**  N  n (%) | 0  0 | 0  0 | 1  1 (100.0) | 0  0 | 0  0 | 0  0 | 1  1 (100.0) |
| **50 µg/kg**  N  n (%) | 7  2 (28.5) | 3  1 (33.3) | 6  4 (66.6) | 3  2 (66.6) | 3  2 (66.6) | 2  1 (50.0) | 6  4 (66.6) |
| **75 µg/kg**  N  n (%) | 9  0 | 5  1 (20.0) | 7  2 (28.6) | 1  1 (100.0) | 1  1 (100.0) | 1  1 (100.0) | 5  3 (60.0) |
| **100 µg/kg**  N  n (%) | 2  1 (50.0) | 1  0 | 0  0 | 0  0 | 0  0 | 0  0 | 1  0 |

ADA, anti-drug antibody; C1D23, Cycle 1 Day 23; C2D1, Cycle 2 Day 1; C3D1, Cycle 3 Day 1; C4D1, Cycle 4 Day 1; C5D1, Cycle 5 Day 1; EOS, End of Study.

This table presents additional data for immunogenicity, specifically by summarizing anti-drug antibody response by dose level and timepoint.

**Supplementary Figure 1.**


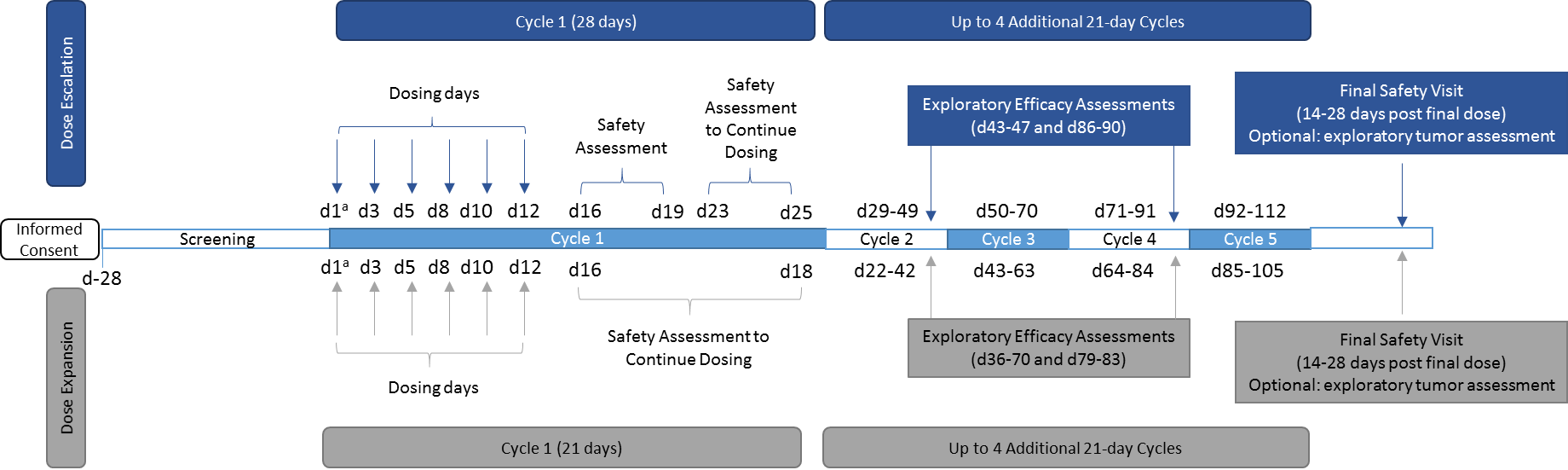


**Figure S1. Study Design for Dose Escalation and Dose Expansion.** Study timeline, including dosing scheme, assessment schedule, and safety visit plan.

^a^Baseline visit.

This figure is a linear visualization of the study scheme. This includes study activities related to enrollment, dosing, and assessments for both safety and efficacy.

**Supplementary Figure 2.**


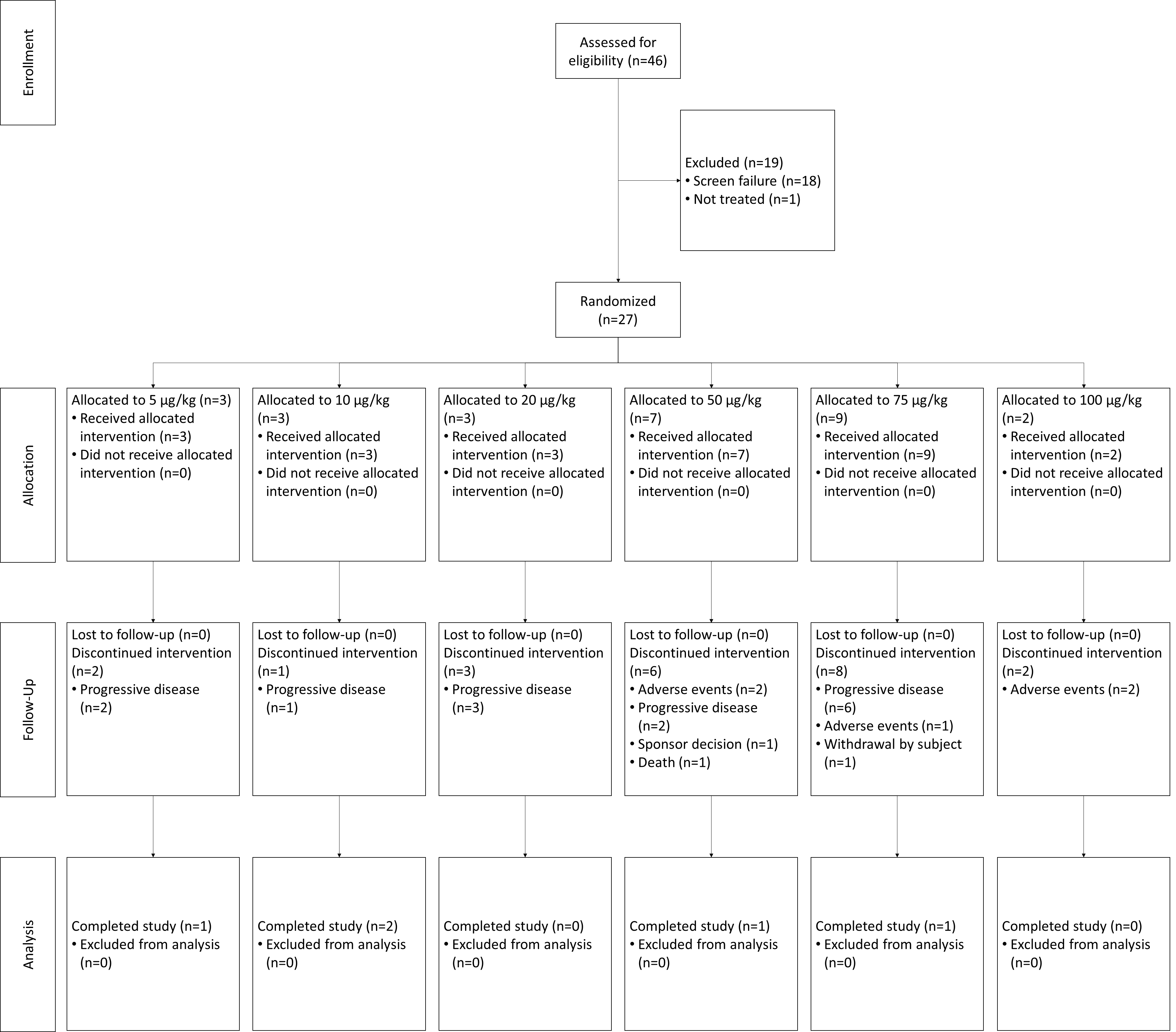


**Figure S2. CONSORT diagram.** Study enrollment, allocation to dose-level, follow-up, and data analysis.

This figure is a visualization of the CONSORT diagram that guided subject enrollment, allocation, follow-up, and data analysis. Total number of subjects is included in each diagram step.

**Supplementary Figure 3.**

**Figure S3. Individual CD19+ Nadir Percent Change from Baseline.**

^a^CD19+ nadir: the minimum observed percent change from baseline for any sample after baseline.

^b^Baseline: the value observed at screening. If no screening value was available, the Cycle 1 Day 8 value was considered as baseline.

Anti-drug antibody (ADA) and neutralizing antibody (Nab) levels for patients with (D) diffuse large B-cell lymphoma and/or (F) follicular lymphoma with baseline CD19+ >10 cells/µL and with at least three CD19+ assessments after baseline.

This figure presents the individual subject data for the minimum observed percent change in CD19+ from baseline for any sample taken after baseline (the value observed at screening).
